# Bruceine D induces lung cancer cell apoptosis and autophagy via the ROS/MAPK signaling pathway in vitro and in vivo

**DOI:** 10.1038/s41419-020-2317-3

**Published:** 2020-02-18

**Authors:** Jiangjiang Fan, Dongmei Ren, Jinxia Wang, Xiaoqing Liu, Huaran Zhang, Mingsheng Wu, Guotao Yang

**Affiliations:** 1 Department of Thoracic Surgery, Qilu Hospital of Shandong University, 107 West Wenhua Road, Jinan, 250012 P. R. China; 2 Key Laboratory of Chemical Biology (Ministry of Education), School of Pharmaceutical Sciences, Shandong University, 44 West Wenhua Road, Jinan, 250012 P. R. China

**Keywords:** Non-small-cell lung cancer, Autophagy, Toxicology

## Abstract

Worldwide, lung cancer remains a leading cause of cancer mortality. Bruceine D (BD) has been shown to induce pancreatic cancer cell death via several different mechanisms. In this study, we demonstrated that BD inhibited lung cancer cell proliferation. Apoptosis and autophagy were the most important mechanisms involved in BD-induced lung cancer cell death, and complete autophagic flux was observed in A549 and NCI-H292 cells. In addition, BD significantly improved intracellular reactive oxygen species (ROS) levels. BD-mediated cell apoptosis and autophagy were almost inhibited in cells pretreated with N-acetylcysteine (NAC), an ROS scavenger. Furthermore, MAPK signaling pathway activation contributed to BD-induced cell proliferation inhibition and NAC could eliminate p-ERK and p-JNK upregulation. Finally, an in vivo study indicated that BD inhibited the growth of lung cancer xenografts. Overall, BD is a promising candidate for the treatment of lung cancer owing to its multiple mechanisms and low toxicity.

## Introduction

Lung cancer is the leading cause of cancer incidence death, with a 5-year survival rate of 18%^1^. Although new diagnostic techniques, chemotherapeutic agents and therapeutic strategies have been developed for cancer treatment, the disease remains a critical challenge due to drug resistance, metastasis and low long-term survival rates^2,3^.

The seeds of *Brucea javanica* have been used to treat inflammation, malaria, and warts for many years^4^. Recently, growing evidence has indicated that *Brucea javanica* extracts exhibit potential anticancer activity^5–7^. BD, a quassinoid compound, can be extracted from the seeds of *Brucea javanica*. A previous study reported that BD induced PANC-1 apoptosis via activating the p38-MAPK signaling pathway^8^. In addition, p38-MAPK pathway activation and NF-kappaB inhibition were crucial for BD-mediated pancreatic cell death^9^. Furthermore, BD suppressed hepatocellular carcinoma growth in vivo and in vitro by diminishing Wnt translation activity^10^. Although the antitumor activity of BD has been reported in many studies, the exact mechanisms have not yet been demonstrated, particularly the mechanisms involved in BD-induced lung cancer cell proliferation inhibition.

Autophagy is a cytoplasmic component degradation process that is paramount in cellular homeostasis and survival. The cardinal steps of autophagosomes formation are regulated mainly by series of essential autophagy-related (ATG) proteins^11^. Microtubule-associated protein 1 A/1B-light chain 3 (LC3) is considered to be one of the most important regulators of the autophagy process^12,13^. The precursor of LC3 is cleaved by ATG4B, resulting in the cytosolic isoform LC3-I^14^. Subsequently, phosphatidylethanolamine binds to LC3-I through ubiquitination to form LC3-II response to autophagy inducers^15,16^. Thus, LC3-I and LC3-II transformation acts as an important indicator for assessing autophagy activity^17^. mTOR, a serine/threonine kinase, is a master induction factor of autophagy^18,19^. The direct phosphorylation of mTORC1 by Akt is critical to the activation of mTORC1^20^. The negative regulation of mTOR by AMPK is what links cellular energy and mTORC1 regulation^21^. ULK, a serine/threonine kinase, may function as a bridge between energy sensors (mTOR, AMPK) and autophagosome formation^22^. During autophagy, cytoplasmic materials and organelles are sequestered and engulfed by a double membraned structure, termed the autophagosome, then delivered to the lysosome for degradation and recycling^23,24^. Autophagy is a non-apoptotic cell death and overwhelming evidence has elucidated the complex relationship between apoptosis and autophagy, the relationship apoptosis and autophagy is unrevealed^25,26^.

The accumulation of reactive oxygen species (ROS) plays an important role in apoptosis and autophagy^27,28^. A recent study suggested that ROS can inhibit MAPK/ERK signaling decreasing the release of epidermal growth factor (EGF)^29^. Furthermore, EGFR phosphorylation and the levels of its downstream effector phosphorylated ERK were markedly inhibited in these cells via a ROS-dependent pathway^30^. Therefore, ROS mediate the MAPK signaling pathway and may be useful for cancer therapy.

In the present study, we explored BD-induced anticancer activity in vitro and in vivo. Our results indicated that BD significantly suppressed A549 and NCI-H292 cell proliferation. Moreover, the activation of ROS-mediated MAPK pathways is essential for BD-mediated apoptosis and autophagy.

## Results

### BD structure

**Fig. 1:**
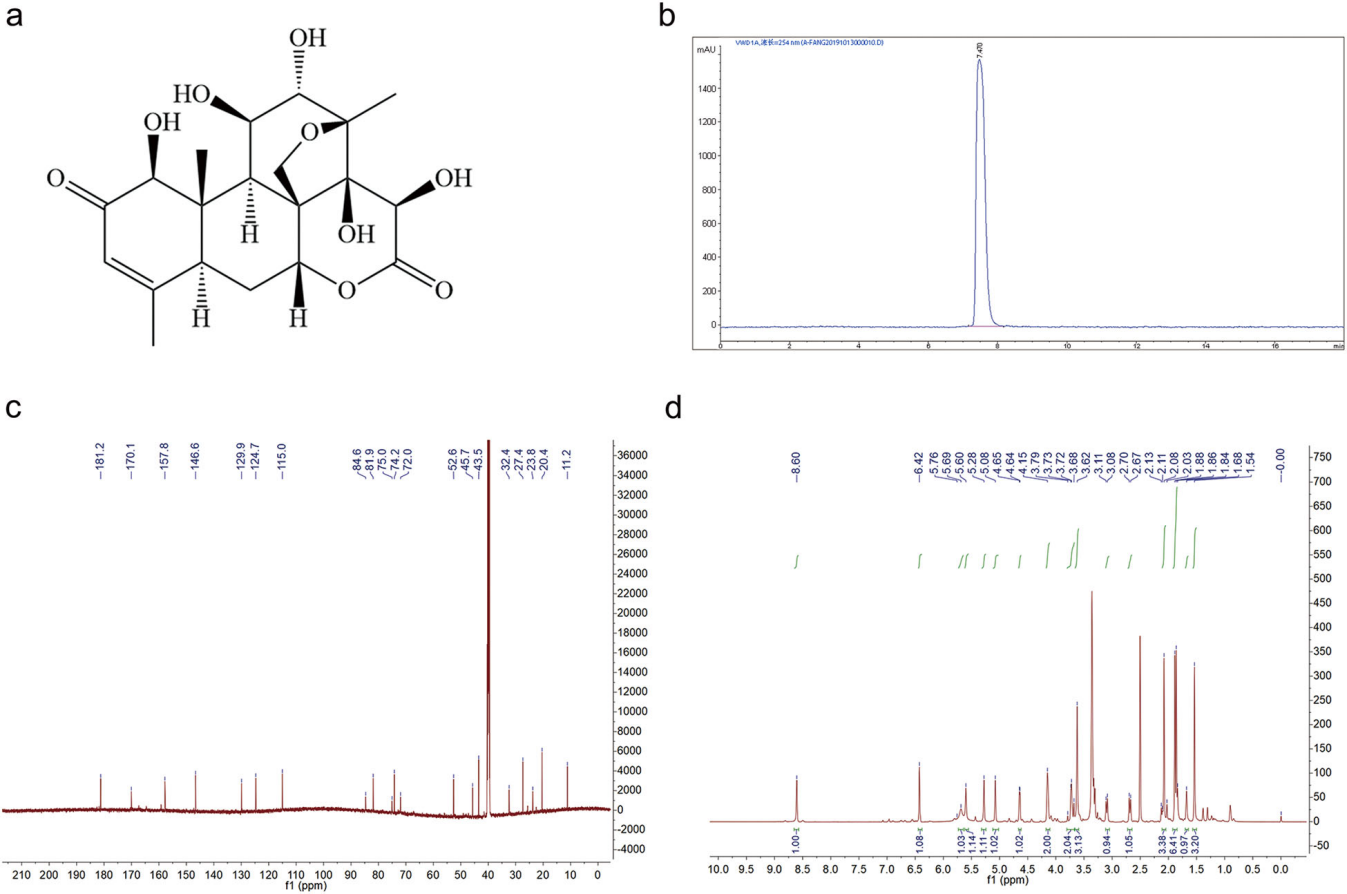
Structures of BD. **a** Chemical structure of BD. **b** HPLC chromatogram. **c** ^13^C NMR spectrum of BD. **d** ^1^H-NMR spectrum of BD.

^1^H and ^13^C nuclear magnetic resonance (NMR) was used to identify the structure of BD (Fig. 1c, d). HPLC analysis was performed in our study to confirm that BD had a purity of >99% (Fig. 1b). ^13^C NMR (150 MHz, DMSO-*d*_6_): 198.8 (C-2), 174.2 (C-16); 163.8 (C-4), 124.5 (C-3); 22.5 (C-18), 11.4 (C-19), 18.7 (C-22). ^1^H NMR (600 MHz, DMSO-*d*_6_) δ ppm: 1.05 (3 H, s), 1.25 (3 H, s) 1.90 (3 H, s), 5.99 (1 H, s, H-3), 2.91 (1 H, d, *J* = 12.7 Hz, H-5), 2.16 (1 H, d, *J* = 14.3 Hz, H-6α), 1.71 (1 H, t, *J* = 13.8 Hz, H-6β), 5.17 (1 H, s, H-7), 2.34 (1 H, s, H-9), 4.92 (1 H, s, H-11), 4.13 (1 H, m, H-12), 4.97 (1 H, d, *J* = 5.0 Hz, Ha-20), 3.70 (1 H, d, *J* = 5.0 Hz, Hb-20).

### BD inhibits the proliferation of A549 and NCI-H292 lung cancer cells in vitro

**Fig. 2:**
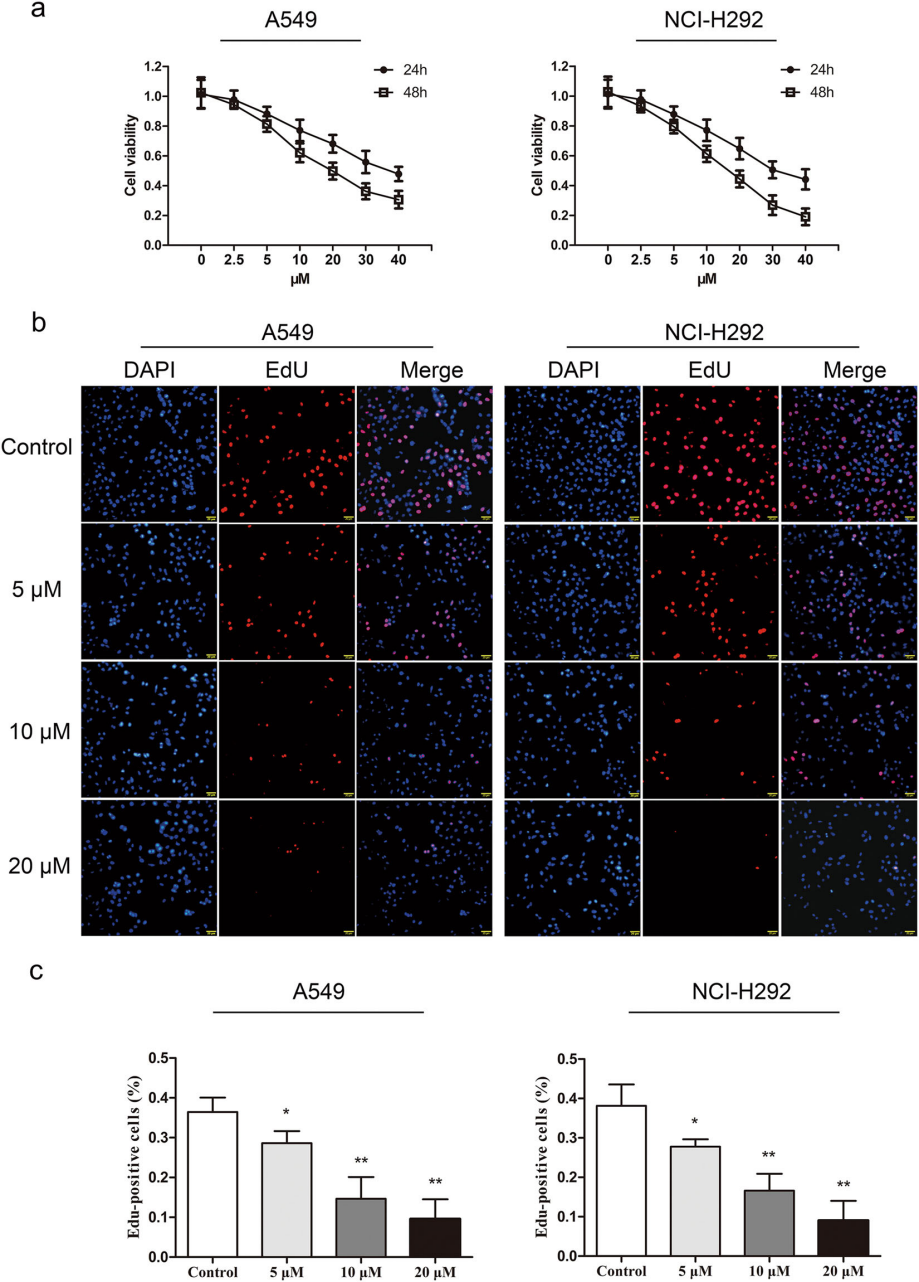
BD inhibited cell proliferation in A549 and NCI-H292 cell lines. **a** Two human lung cancer cell lines (A549 and NCI-H292) were treated with BD (2.5, 5, 10, 20, 30, and 40 µM) for 24 h and 48 h. The cell viability was evaluated by CCK-8. **b** A549 and NCI-H292 cells were treated with BD for 48 h. Then cells were stained with Azide 594 (red) to detect EdU and DAPI (blue) to stained nuclei. Fluorescence images were obtained and analyzed with a fluorescence microscope. **c** Values are expressed as the mean ± SD, *n* = 3 *p < 0.05, **p < 0.01, and ***p < 0.001 compared to control groups.

A549 and NCI-H292 cells were exposed to different concentrations of BD for 24 h and 48 h. A CCK-8 assay was used to detect cell proliferation and viability. As shown in Fig. 2a, BD inhibited human lung cancer cell growth in a dose- and time-dependent manner. The IC50 (24 h) values for A549 and NCI-H292 cells were 36.76 and 31.22 μM, respectively, and the IC50 (48 h) values were 17.89 and 14.42 μM, respectively. In addition, BD did not show apparent toxicity in human umbilical vein cells EA.hy926 or HUVECs (Fig. S1a, b).

Quantitative analysis of EdU incorporation indicated that BD (20 μM) markedly inhibited the proliferation of A549 and NCI-H292 cells (Fig. 2b, c). These results provide further evidence that BD potentially inhibits lung cancer cell proliferation in a dose-dependent manner.

### BD induces mitochondria-dependent apoptosis in A549 and NCI-H292 cells

**Fig. 3:**
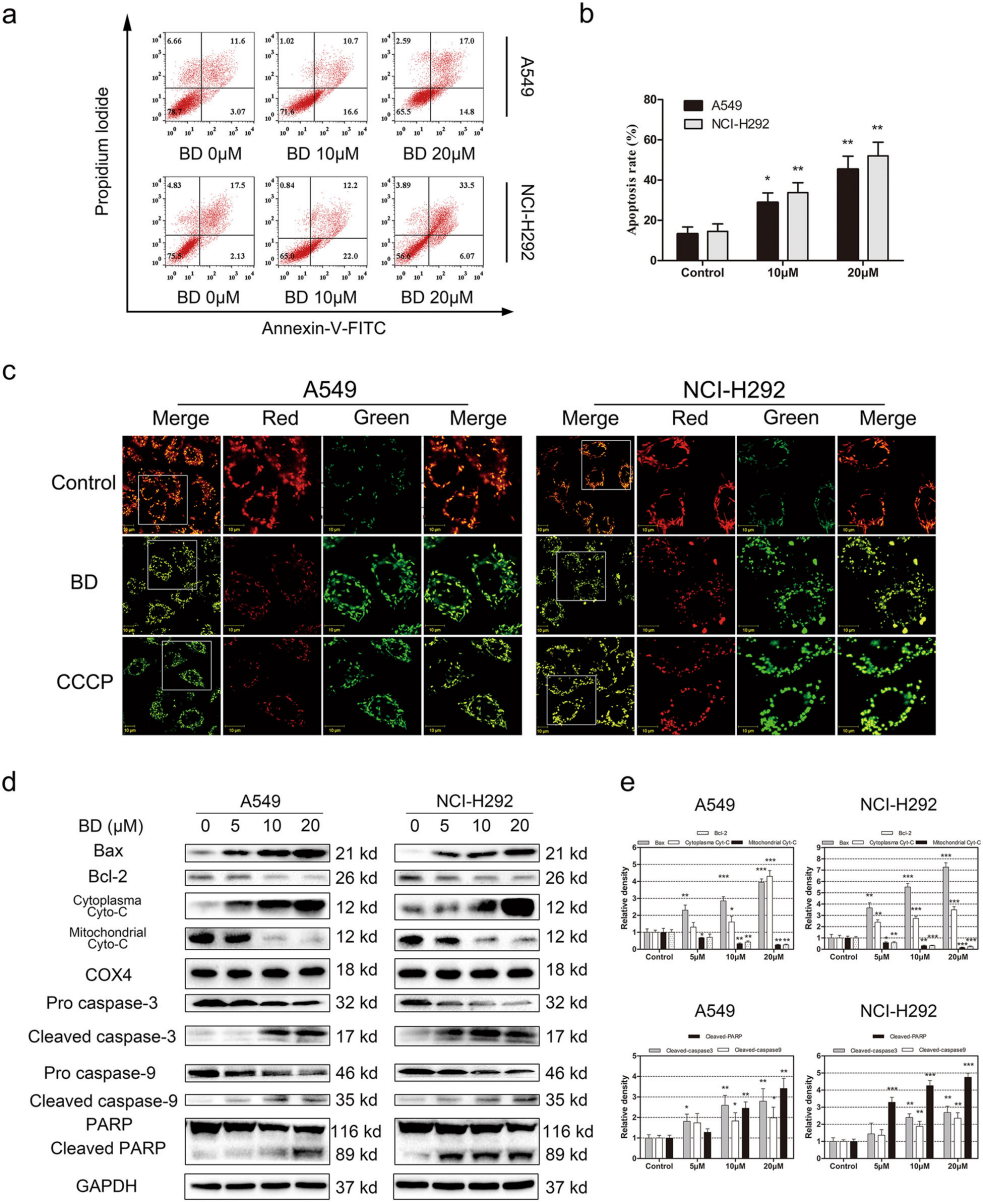
BD inhibited mitochondria-dependent apoptosis in A549 and NCI-H292 cells. **a** A549 and NCI-H292 cells were treated with BD (10 and 20 μM) for 48 h and stained with annexin v-FITC/PI and analyzed by flow cytometry. **b** Quantification of apoptotic cells induced by BD. Values are expressed as the mean ± SD, *n* = 3 *p < 0.05, **p < 0.01, and ***p < 0.001 compared to control groups. **c** The changes of MMP were monitored by JC-1 fluorescent probe and observed by confocal microscopy. **d** The expression of apoptosis-related proteins were determined by western blotting. **e** Histogram represented the relative expression level of apoptosis-related proteins. Values are expressed as the mean ± SD, *n* = 3 *p < 0.05, **p < 0.01, and ***p < 0.001 compared to control groups.

Annexin V-FITC and PI double staining was performed to validate that cell apoptosis is involved in the cytotoxic effects of BD. As shown in Fig. 3a, b, the proportion of apoptotic cells increased after treatment with different concentrations of BD. In addition, compared with the control conditions, BD potentially increased the early apoptosis rate in A549 cells and late apoptosis rate in NCI-H292 cells. MMP depolarization is the final crucial step of the early stage of cell apoptosis. The JC-1 staining results indicated that the red/green ratio decreased after BD treatment, and carbonyl cyanide 3-chlorophenylhydrazone (CCCP) was used as a positive control (Fig. 3c). Quantitative studies also confirmed the MMP collapse (Fig. S2a, b). Furthermore, Western blotting was used to investigate the expression of apoptotic related proteins. As shown in Fig. 3d, e, BD induced apoptosis by upregulating Bax expression and decreasing the Bcl-2/Bax ratio and cytochrome *c* expression in the mitochondria. Cleaved caspase-9, cleaved caspase-3, and cleaved PARP and cytochrome *c* upregulation in the cytoplasm was observed. These results revealed that BD induced mitochondria-dependent apoptosis in lung cancer cells.

### BD induces autophagy in lung cancer cells

**Fig. 4:**
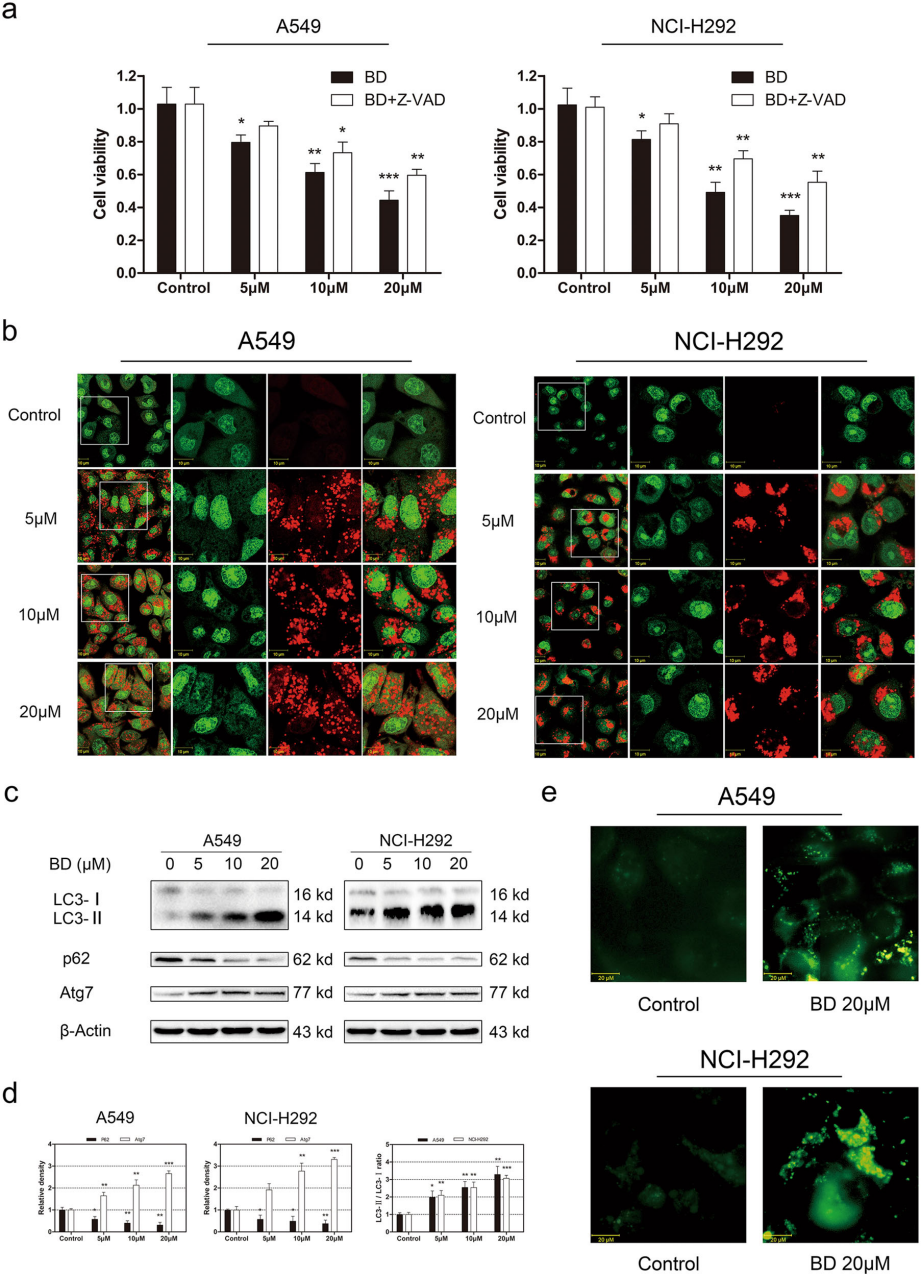
BD induces autophagy in A549 and NCI-H292 cells. **a** Effect of pre-treatment with z-VAD-fmk (20 μM) on A549 and NCI-H292 cell survival was evaluated by CCK-8 assay. **b** A549 and NCI-H292 cells were treated with BD for 24 h and stained with acridine orange. AO-stained cells were observed by confocal microscopy. **c** A549 and NCI-H292 cells were treated with BD (5, 10, and 20 μM) for 24 h and the expression of LC3, p62, and Atg7 was assessed by western blot. **d** Values are expressed as the mean ± SD, *n* = 3 *p < 0.05, **p < 0.01, and ***p < 0.001 compared to control groups. **e** A549 and NCI-H292 cells were infected with GFP-LC3-expressing vectors and then exposed to BD. GFP-LC3 punctae were acquired by fluorescent microscopy.

**Fig. 5:**
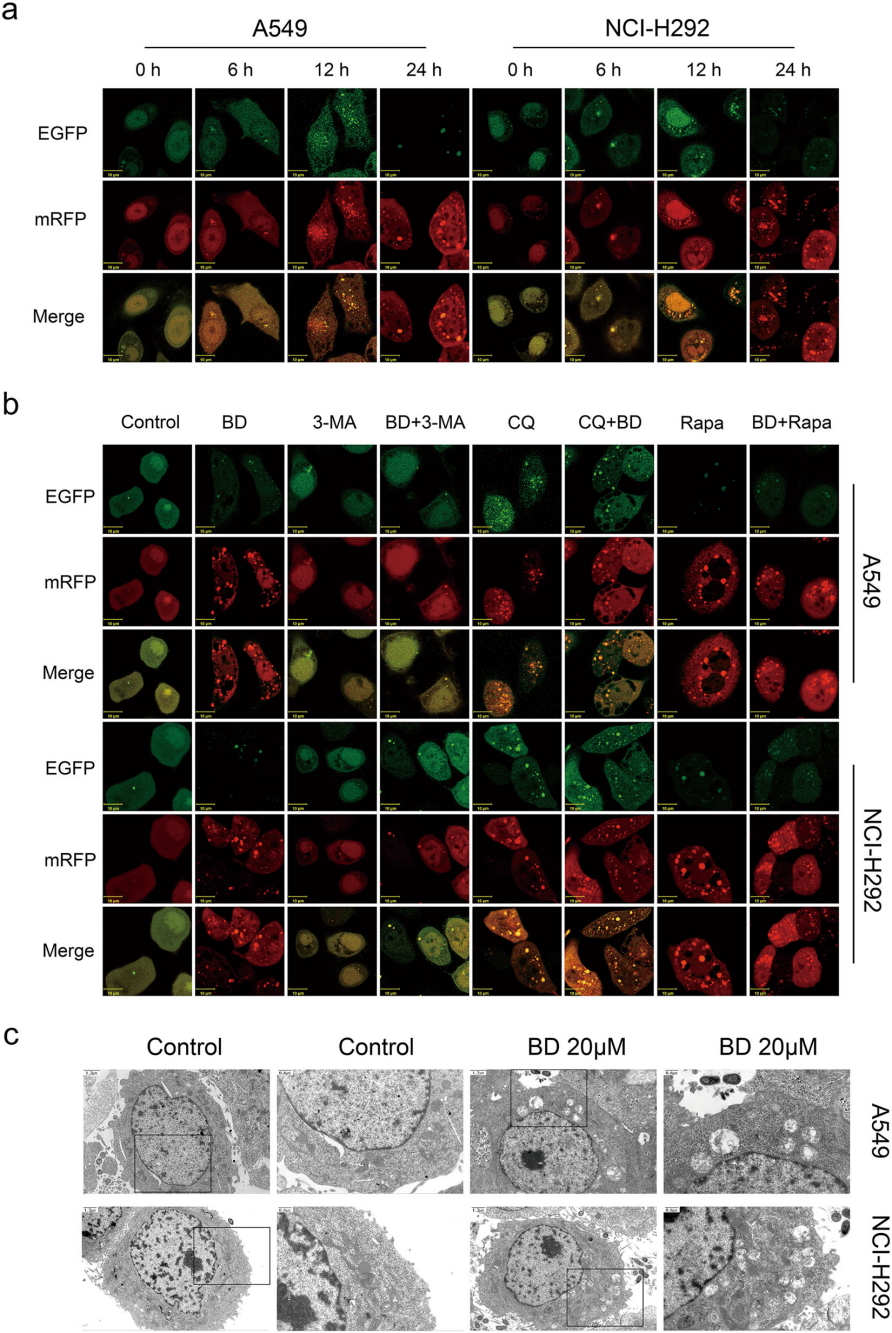
BD induces autophagic flux in A549 and NCI-H292 cells. **a** A549 and NCI-H292 cells overexpressing mRFP-GFP-LC3 were treated with 20 μM BD for the indicated times and then subjected to confocal microscopy. Scale bar: 10 μm. **b** Cells overexpressing mRFP-GFP-LC3 were treated with BD, Rapa, 3-MA and CQ for the indicated times and then observed under confocal microscopy. **c** The formation of autophagosome was observed in transmission electron microscopy, The scale bar in the original image and the enlarged image represents 0.6 μm and 1.2 μm, respectively.

The irreversible caspase inhibitor z-VAD-fmk was used to further confirm the mechanism by which apoptosis is involved in BD-induced proliferation inhibition. Nevertheless, the inhibited cell viability induced by BD was partly reversed by z-VAD-fmk (Fig. 4a), which means that other mechanisms are likely also involved. We observed red acidic vesicle accumulation under fluorescece microscopy after staining with AO (Fig. 4b). In addition, we investigated the expression of autophagy-related proteins upon BD treatment. Western blot results revealed that LC3-II/LC3-I ratio was strongly increased and that the P62 level was decreased in a dose-dependent manner (Fig. 4c, d). Atg7, another key indicator of autophagy, was upregulated with BD treatment. To further address the role of BD-induced autophagy in lung cancer cells, we generated stable GFP-LC3-expressing lung cancer cells. The percentage of GFP-LC3-positive puncta was increased in BD-treated cells (Fig. 4e). TEM images can directly indicate autophagosome formation. As shown in Fig. 5c, massive intracellular autophagosome vacuoles accumulated upon treatment with BD. As a result, the present study showed that BD induced autophagy in human lung cancer cells.

### BD promotes autophagy flux in lung cancer cells

To dynamically visualize LC3-labelled cytoplasmic vacuolation to further clarify whether the complete progression of autophagy was affected by BD, mRFP-GFP-LC3 adenovirus was transfected into A549 and NCI-H292 cells. Compared with the relatively stable mRFP signal, GFP is more sensitive to acidic lysosomes. As shown in Fig. 5a, A549 and NCI-H292 cells treated with BD (20 µM) for 12 h had slight detectable yellow autophagic LC3 puncta (mRFP+/GFP+) and red autophagic LC3 puncta (mRFP+/GFP−) accumulation in the cytoplasm compared with untreated control cells. More importantly, red puncta (mRFP+GFP−) containing mRFP-LC3 became predominantly visible, but not green puncta (mRFP−GFP+), in cells after 24 h of BD treatment. This result suggests that BD treatment can induce both autophagosome and autolysosome accumulation, consistent with the Western blotting results.

A549 and NCI-H292 cells were transfected with mRFP-GFP-LC3 adenovirus and exposed to BD for 24 h. Rapamycin (Rapa) was used as a positive control, and 3-MA and chloroquine (CQ) were used for autophagic flux detection. As shown in Fig. 5b, Rapa could enhance BD-induced autolysosomes (red), and CQ could induce yellow autophagic LC3 puncta (mRFP+/GFP+) accumulation. In addition, the number of red puncta induced by BD was reduced by 3-MA. Collectively, our confocal microscopy images demonstrated that BD promotes autophagy flux in lung cancer cells.

### ROS accumulation and MAPK signaling pathway regulation are involved in BD-induced proliferation inhibition

**Fig. 6:**
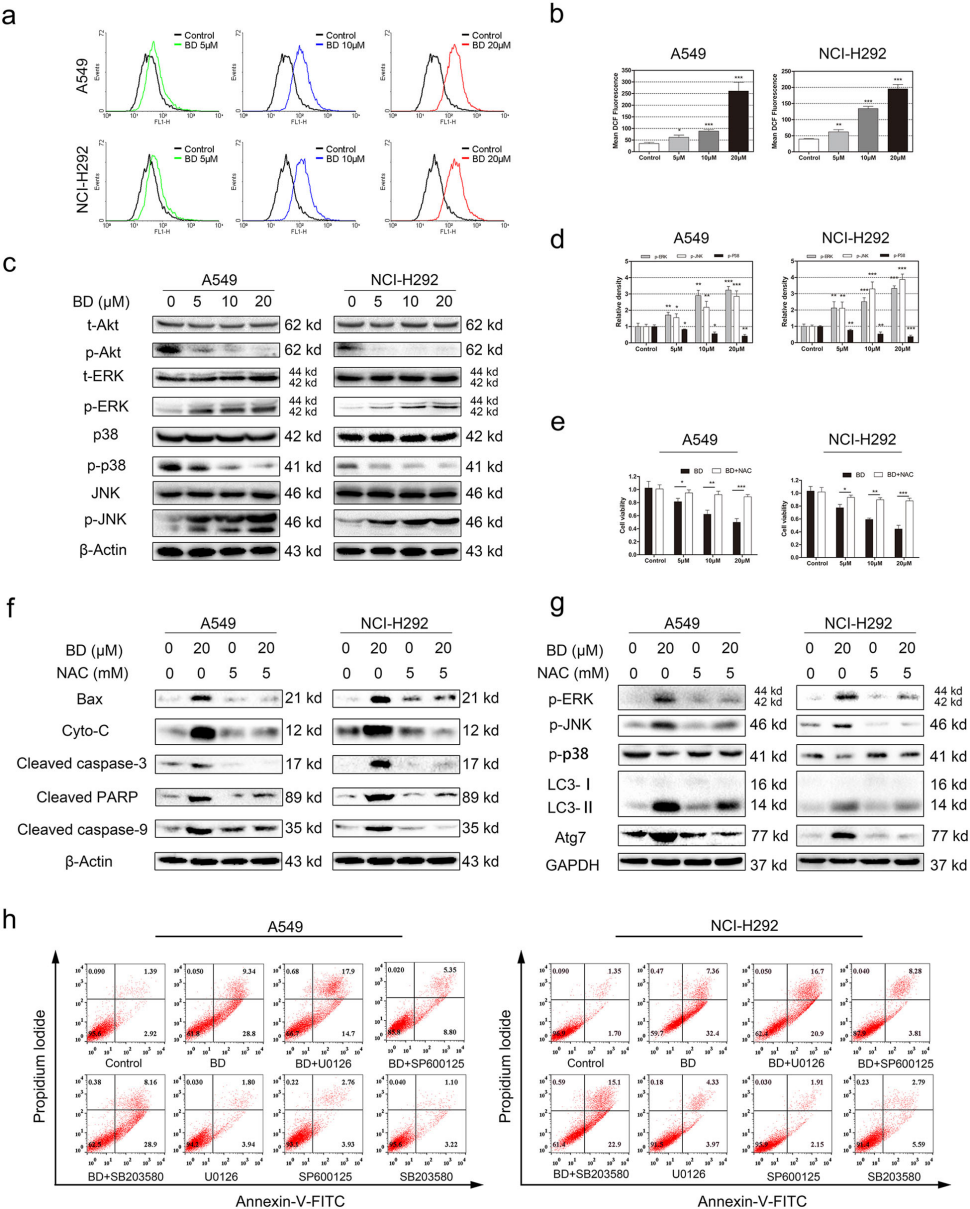
MAPK activation is triggered by BD and roles of ROS in autophagy and apoptosis induced by BD. **a** A549 and NCI-H292 cells were treated with various concentrations of BD for 24 h and the level of ROS was determined by flow cytometry. **b** Quantitative analysis of the fluorescent intensity is shown in histograms. Values are expressed as the mean ± SD, *n* = 3 *p < 0.05, **p < 0.01, and ***p < 0.001 compared to control groups. **c** A549 and NCI-H292 cells were treated with BD (5, 10, and 20 μM) for 24 h and the expression of p-ERK, t-ERK, p38, p-p38, JNK, p-JNK, p-Akt, and t-Akt was assessed by western blot. **d** Values are expressed as the mean ± SD, *n* = 3 *p < 0.05, **p < 0.01, and ***p < 0.001 compared to control groups. **e** A549 and NCI-H292 cell viability was assessed by CCK-8. **f** A549 and NCI-H292 cells were pretreated with NAC for 2 h and treated with various concentrations of BD. The expression of Bax, cytochrome *c*, cleaved caspase-3, cleaved PARP and cleaved caspase-9 was assessed by western blot. **g** The expression of p-ERK, p-JNK, p-p38, LC3, and Atg7 was assessed by western blot. **h** Cells were pretreated with U0126, sp600125, SB203580 for 2 h and exposed to BD for 48 h, then stained with annexin v-FITC/PI and analyzed by flow cytometry.

ROS generation is strongly associated with the regulation of apoptosis. Our flow cytometry results revealed that BD induced ROS production (Fig. 6a, b). In addition, BD upregulated the expression of p-ERK and p-JNK while decreasing phospho-p38 protein expression (Fig. 6c, d). The CCK-8 results indicated that NAC, an antioxidant, rescued cell death (Fig. 6e). The upregulation of apoptosis-related proteins induced by BD was diminished by NAC (Fig. 6f). Furthermore, NAC abolished the phosphorylation of ERK and JNK (Fig. 6g). To verify which kinase is essential for BD-induced apoptosis, U0126 (a MEK inhibitor), SP600125 (a JNK inhibitor), and SB203580 (a p38 inhibitor) were used in our apoptosis experiment. As shown in Fig. 6h, SP600125 suppressed BD-induced apoptosis. A western blot assay was used to assess ROS generation in BD-induced autophagy (Fig. 6g). These results suggested that ROS generation may serve as a critical initial mediator of BD-induced apoptosis and autophagy.

### z-VAD-fmk enhances autophagy, while CQ abolishes apoptosis induced by BD

**Fig. 7:**
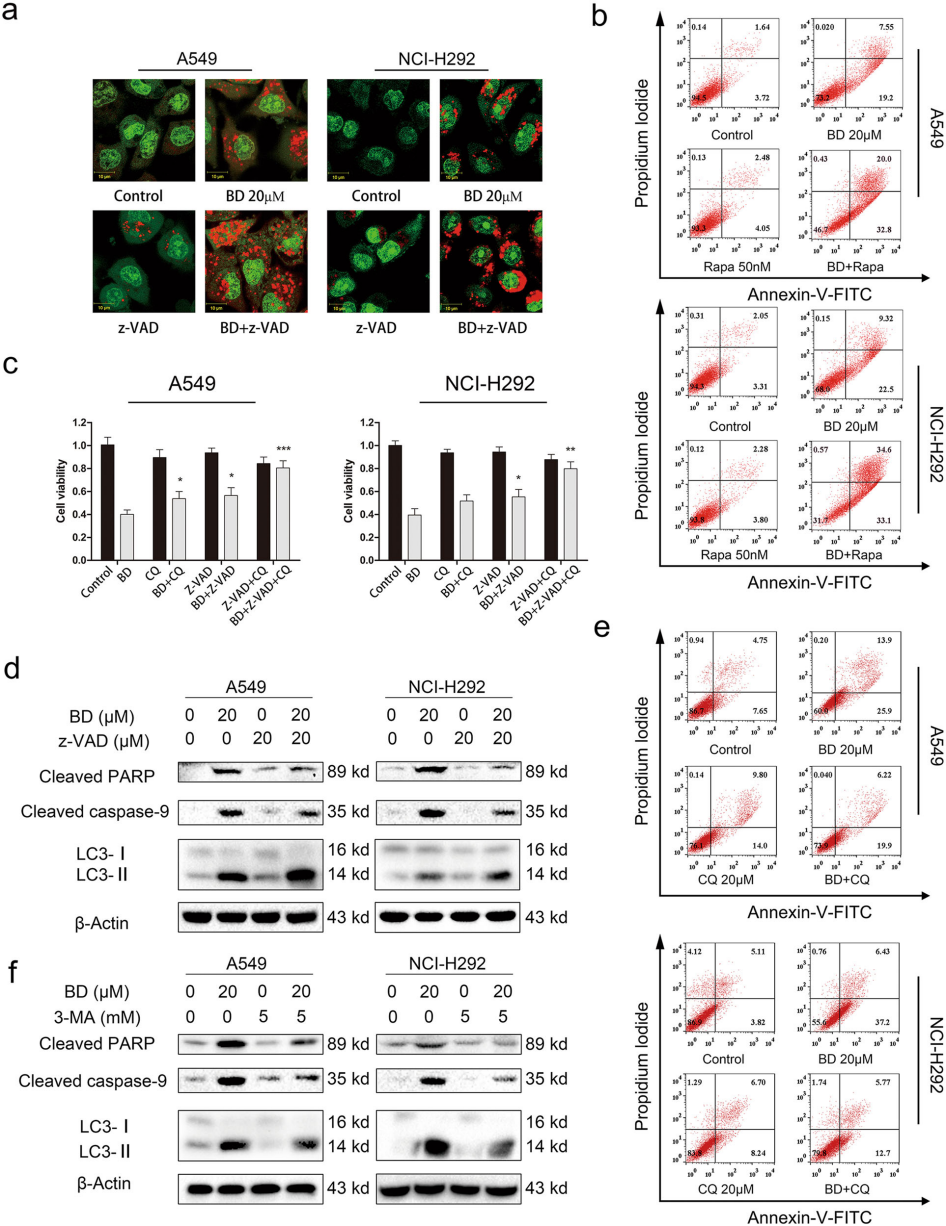
The relationship between apoptosis and autophagy. **a** Acridine orange-stained cells were observed by confocal microscopy. **b** A549 and NCI-H292 cells were pretreated with Rapa (50 nM) and then treated with BD for 48 h. Then cells were stained with annexin v-FITC/PI and analyzed by flow cytometry. **c** A549 and NCI-H292 cells were pretreated with CQ (20 μM) or z-VAD-fmk (20 μM) for 2 h and then treated with BD (20 μM) for 24 h. CCK-8 assay was used to evaluate cell viability. **d** Cells were pretreated with z-VAD-fmk (20 μM) for 2 h and then treated with BD for 24 h. The expression of cleaved caspase-9, LC3 and cleaved PARP was assessed by western blot. **e** A549 and NCI-H292 cells were pretreated with CQ (20 μM) and then treated with BD for 48 h. Then cells were stained with annexin v-FITC/PI and analyzed by flow cytometry. **f** Cells were pretreated with 3-MA (5 mM) and then treated with BD for 24 h. The expression of cleaved caspase-9, LC3, and cleaved PARP was assessed by western blot.

The relationship between apoptosis and autophagy was also investigated in our study. The CCK-8 assay results showed that the combination of BD with CQ or z-VAD-fmk could mitigate the BD-induced cell proliferation inhibition (Fig. 7c). Then, z-VAD-fmk, CQ, and 3-MA were used to determine the association between apoptosis and autophagy. Western blotting results demonstrated that z-VAD-fmk and 3-MA could decrease the expression of cleaved caspase-9 and cleaved PARP (Fig. 7d, f). The fluorescence results were consistent with those of the CCK-8 assay, revealing that z-VAD-fmk could slightly promote LC3-II expression (Fig. 7d) and red acidic vesicle accumulation induced by BD (Fig. 7a). As shown in Fig. 7b, e, 3-MA could enhance BD-induced apoptosis, and CQ could partly block BD-induced apoptosis.

### BD inhibits the growth of lung cancer in vivo

**Fig. 8:**
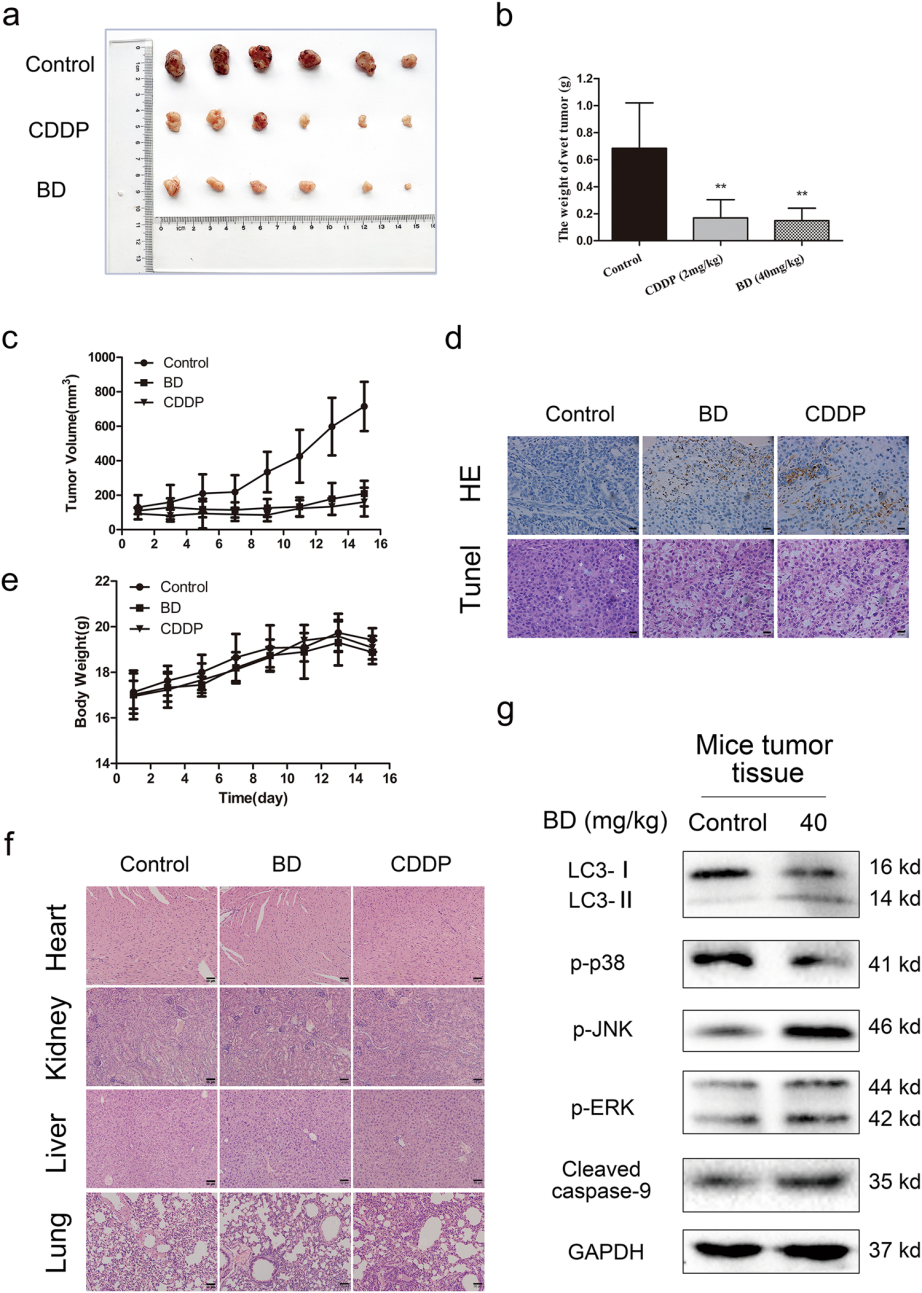
BD inhibits the growth of lung cancer xenografts in vivo. **a** A549 cells were inoculated in BALB/c-nu mice. Once the tumor volume reached around 100 mm^3^, mice were randomly allocated into three groups for treatment control group (saline i.p), BD group (40 mg/kg i.p) and CDDP group (2 mg/kg i.p). **b** BD significantly inhibited the growth of tumor xenografts. **c** Tumor volume was measured every two days. **d** H&E staining and TUNEL assay were performed to examine histological morphology and apoptosis of tumor tissues. **e** Body weights were measured every 2 days. **f** H&E staining results showed no significant organ related toxicities. The expression of p-p38, p-JNK, p-ERK, LC3, and cleaved caspase-9 was assessed by western blot.

A tumor xenograft mouse model was used to assess the anticancer activity of BD in vivo. Tumor-bearing mice received i.p. injections of saline, BD (40 mg/kg) and CDDP (2 mg/kg) for 15 days. As shown in Fig. 8a, b, c and e, BD significantly reduced the growth of xenograft tumors without significant changes in body weight. We observed an increase in the number of TUNEL-positive cells and upregulation of LC3-II, cleaved caspase-9 p-ERK and p-JNK following BD treatment (Fig. 8d, g). H&E staining of several of the most important organs was performed to evaluate the cytotoxic effects of BD. As shown in Fig. 8f, the H&E staining results suggested that BD exhibited no significant toxicity in vivo.

## Discussion

Searching for chemotherapeutic adjuvant drugs that work through various anticancer mechanisms may lead to new targeted agents and therapeutic strategies. In this study, we found that BD, a quassinoid compound, has potential anticancer activity in A549 and NCI-H292 cells. Our results reveal that BD induces lung cancer cell apoptosis and autophagy, which is regulated by ROS generation and the MAPK signaling pathway.

Apoptosis is characterized by cell shrinkage, chromatin condensation, nuclear fragmentation, plasma membrane blebbing, and debris clearance by neighbouring phagocytes^31,32^. In our study, BD exhibited strong anticancer activity and inhibited proliferation. In further experiments, we found that BD could induce cell apoptosis and that this type of pro-apoptotic activity cannot be entirely blocked by z-VAD-fmk. TEM results revealed that BD induced autophagy and facilitated autophagic flux in lung cancer cells. Moreover, BD could cause ROS overproduction and regulate apoptosis and autophagy via the MAPK signaling pathway. Our results indicated that Inhibiting apoptosis promoted autophagy, while suppressing of autophagy diminished cell apoptosis caused by BD.

ROS, small and highly reactive molecules, are the normal metabolite products of mitochondria, and ROS overproduction serves as an important mediator in cell death in response to various stimuli^33–35^. Accumulating evidence has indicated that excessive ROS generation contributes to cell cycle arrest and mitochondria-dependent apoptosis^36,37^. Our results revealed that BD strongly induced ROS overproduction, MMP depolarization, cytochrome *c* release, caspase cascade activation, and PARP cleavage. More importantly, BD-mediated apoptosis was almost entirely reversed by NAC.

Autophagy, a conserved catabolic process, is characterized by double membrane autophagosome formation^38,39^. Growing evidence has confirmed that suppressing autophagy enhances therapeutic efficacy^40,41^. LC3 is the most widely detected protein marker and is considered to be reliably associated with completed autophagosomes^17,42^. In addition, the delivery of ubiquitin-tagged substrates to autophagosomes and lysosomes is modulated by p62^43^. Recent research has demonstrated that Atg7 is essential for autophagy flux^44^. We observed that BD could increase the LC3-II/LC3-I ratio, downregulate p62 expression and promote autophagosome formation. Moreover, a dual fluorescent tag indicated that BD facilitated autophagy flux.

Although many studies have focused on the intricate relationship between apoptosis and autophagy, the mechanism is still undetermined^45^. We observed that z-VAD-fmk merely reversed BD-induced cell death and that CQ could partly rescue BD-mediated proliferation inhibition. Growing evidences indicates that ROS accumulation plays an essential role in cell survival, cell death, and autophagy activation^46,47^. In our study, NAC almost abolished BD-induced expression of autophagy- and apoptosis-related proteins.

The MAPK signaling pathway is downstream of ROS and plays an essential role in the induction of apoptosis and autophagy^48^. Many studies have demonstrated that ROS accumulation could induce cell death through MAPK activation^49,50^. Western blot results indicated that apparent increases in the phosphorylation levels of ERK and JNK and NAC almost abolished this effect mediated by BD.

## Materials and methods

### Reagents

*Brucea javanica* seeds were purchased from the Bozhou Chinese herbal medicine market. BD was extracted from the seeds of *Brucea javanica* (Supplementary Information). Cell Counting Kit-8 (CCK-8; CK04–500, Dojindo, Kumamoto, Japan), 2′,7′-dichlorodihydrofluorescein diacetate (DCFH-DA, Sigma), an Annexin V-FITC/propidium iodide (PI) kit (BD Biosciences, San Jose, CA), cisplatin (CDDP, Sigma-Aldrich, St. Louis, MO), dimethyl sulfoxide (DMSO, Solarbio, China), carbonyl cyanide 3-chlorophenylhydrazone (CCCP, Beyotime, China), N-acetylcysteine (NAC, Sigma), acridine orange (AO, Solarbio, China), and 5-ethynyl-2′-deoxyuridine (EdU, Beyotime, China) were purchased from the indicated suppliers. Primary antibodies for Bax, Bcl-2, caspase-9, cleaved caspase-9, caspase-3, cleaved caspase-3, cytochrome *c*, COX4, and GAPDH were purchased from Proteintech Group (Wuhan, China). Akt, p-Akt, p-ERK1/2, JNK, p-JNK, and β-actin antibodies were purchased from Santa Cruz Biotechnology (Dallas, TX). t-ERK1/2, p38, p-p38, p62, LC3, and Atg7 antibodies were purchased from Abcam, and the PARP antibody was purchased from Cell Signaling Technology (Beverly, MA).

### Cell lines and cell culture

A549 and NCI-H292 human lung cancer cell lines and EA.hy926 or HUVECs human umbilical vein cell lines were obtained from the Cell Bank of the Type Culture Collection of the Chinese Academy of Sciences (Shanghai, China). The cells were incubated in RPMI 1640/DMEM (Gibco BRL, Grand Island, NY) with 10% fetal bovine serum, and maintained in a 37 °C incubator with 5% CO_2_.

### Cell viability assay

Cell Counting Kit-8 assays were performed to assess cell viability. Briefly, cells were grown in 96-well plates and incubated for 24 h at a density of 1.0 × 10^4^ cells per well. After treatment with BD for 24 h or 48 h, the cells were incubated for an additional 4 h with 100 μl of RPMI 1640/DMEM and 10 μl of CCK-8 solution at 37 °C. The absorbance of each well was then read at 450 nm using a microplate reader (Bio-Rad, Model 680, USA).

### Cell proliferation assays

An EdU Cell Proliferation Kit with Alexa Fluor 594 was used to evaluate cell proliferation according to the manufacturer’s instructions. In brief, EdU, a thymidine analog, was incorporated into A549 and NCI-H292 cells and detected through the click reaction. The cells were observed by fluorescence microscopy.

### Apoptosis analysis by flow cytometry

Cells (4 × 10^5^) were seeded in 6-well plates, incubated overnight and treated with different concentrations of BD. Forty-8 h after treatment termination, the cells were harvested with trypsin and washed with phosphate-buffered saline (PBS) (3000 rpm, 5 min). Then, an Annexin V-FITC/PI kit was used for cell staining according to the manufacturer’s instructions. Finally, the samples were analyzed by flow cytometry.

### Measurement of intracellular ROS

2′,7′-Dichlorodihydrofluorescein diacetate was used to detect intracellular ROS levels. After incubation with BD for 24 h, the cells were washed with PBS and incubated in fresh medium containing DCFH-DA (10 µg/ml) at 37 °C for 30 min. Then, the cells were collected and analyzed at an excitation wavelength of 488 nm and an emission wavelength of 530 nm by a flow cytometer.

### Detection of acidic vesicular organelles

Autophagy lysosomes, acidic vesicular organelles (AVOs), are a morphological characteristic of autophagy and can be stained with AO. Cells were seeded in confocal dishes and treated with BD for 24 h. After treatment with BD, the cells were stained with AO (10 μg/ml) for 30 min and observed under a laser scanning confocal microscope (LSM 700, Zeiss, Germany).

### Autophagy analysis

A549 and NCIH-292 cells were transfected with mRFP-GFP-LC3 adenovirus (Genechem Co Shanghai, China) and GFP-LC3B-expressing vectors to detect autophagosomes and autolysosomes. The cells were plated in confocal dishes and incubated overnight. After adenovirus transfection for 48 h, the cells were treated with BD. Autophagic flux was observed with a laser scanning confocal microscope (LSM 700, Zeiss, Germany) and fluorescence microscopy.

### Analysis of mitochondrial membrane potential (MMP)

5,5′,6,6′-Tetrachloro-1,1′,3,3′-tetraethyl benzimidazolyl-carbocyanine iodide (JC-1) was used to determine MMP. Cells were seeded in confocal dishes and incubated for 24 h. After treatment with BD, the cells were stained with JC-1 for 45 min at 37 °C. After washing with PBS, we acquired images using a laser scanning confocal microscope (LSM 700, Zeiss, Germany).

### Transmission electron microscopy

Cells were collected and fixed with 2.5% glutaraldehyde at 4 °C overnight at room temperature. Following fixation with 1% osmium tetroxide for 1–2 h, the cells were dehydrated with a series of graded alcohols. After being embedded in epoxy resin, the samples were examined by transmission electron microscopy.

### Mitochondrial extraction

Mitochondria were extracted using a Cell Mitochondria Isolation Kit (Beyotime, China). Cells were seeded in D-100 dishes for 24 h. After incubation with EB30 for another 24 h, the cells were homogenized with mitochondrial extraction reagent and centrifuged at 600 × *g* and 4 °C for 10 min, and the supernatants were centrifuged for an additional 15 min (4 °C, 12,000 g). The resulting pellet sediments contained the mitochondria.

### Western blot analysis

Cell lysates were separated by SDS-PAGE (7–12%) at 120 V and eletrotransferred onto nitrocellulose membranes (Millipore). After blocking with 5% nonfat dry milk in PBS, the membranes were incubated with the primary antibodies at 4 °C overnight and with horseradish peroxidase (HRP)-conjugated secondary antibodies for 1 h at room temperature. GAPDH and β-actin were used as controls. Finally, specific antibody binding was analyzed using Image Lab™ Software on a ChemiDoc XRS + (Bio-Rad, USA).

### Xenograft assays

BALB/c-nu mice (female, 4 weeks) were purchased from Beijing Vital River Laboratory Animal Technology Co, Ltd (Beijing, China). Cells were collected with PBS and mixed with an equal volume of Matrigel at a final concentration of 1 × 10^7^/mL. Then the lung cancer cell suspensions (100 μL) were injected subcutaneously. When the volumes of the tumors grew to approximately 100 mm^3^, the tumor-bearing mice were allocated randomly into three groups: control group (saline i.p), BD group (40 mg/kg i.p) and CDDP group (2 mg/kg i.p) (*n* = 6). The mice were injected with saline, BD and CDDP every 2 days for 15 days. The tumor volumes and body weights were measured every 2 days. The research protocol was performed strictly in accordance with the institutional guidelines of the Animal Care and Use Committee of Shandong University.

### H&E staining

Tissue samples from the mice were fixed with formalin and embedded in paraffin. After cutting into 4 µm sections, the tumor and essential organ specinens were stained with H&E, and histological examinations were performed using an Olympus microscope (Japan).

### TUNEL assay

A TUNEL Assay Kit (Roche) was used to detect apoptosis. The main protocols involve the following steps: deparaffinization, rehydration, postfixation, prehybridization, hybridization, endogenous peroxidase activity inhibition, DAB color development, and counterstaining with hematoxylin. The sections were then dehydrated and mounted with Permount.

### Statistical analysis

The experimental data were analysed using GraphPad Prism software version 7. Two-tailed Student’s *t*-test was utilized to determine significant p-values for comparison of two groups. The results are presented as the mean ± SD from three independent experiments. The variance is similar between the groups. *p < 0.05, **p < 0.01, and ***p < 0.001 were considered statistically significant.

## Supplementary information

Figure S1

Figure S2

Extraction and separation

Reproducibility Checklist

Supplementary figure Legends
