# Supplementary figures and images for: Bruceine D induces lung cancer cell apoptosis and autophagy via the ROS/MAPK signaling pathway in vitro and in vivo
Source: Cell Death Dis. 2020 Feb 18;11(2):126. doi: 10.1038/s41419-020-2317-3 (PMC7028916; doi:10.1038/s41419-020-2317-3)

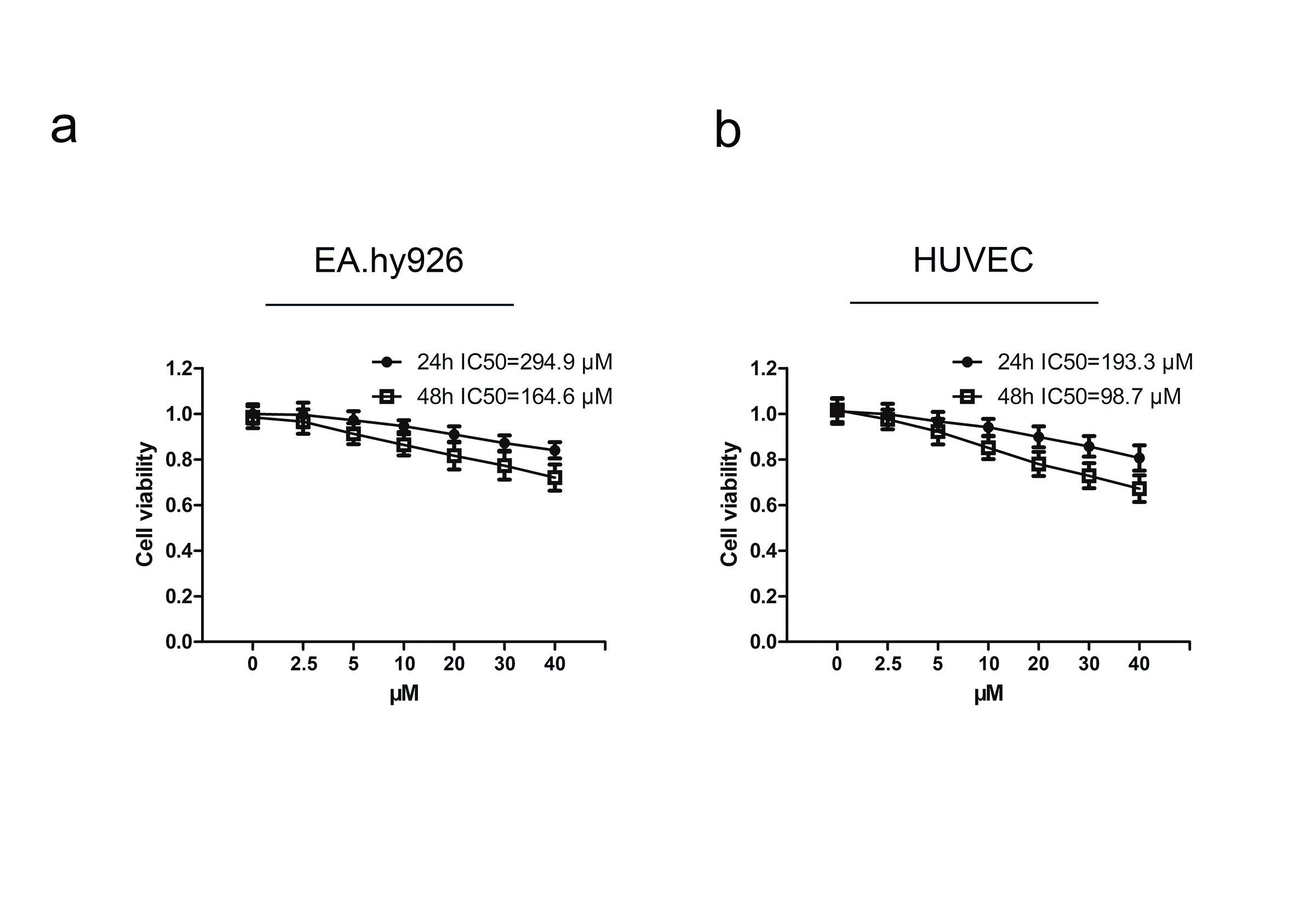

Supplement: Supplementary file 1 — Figure S1 [file 41419_2020_2317_MOESM1_ESM.tif]

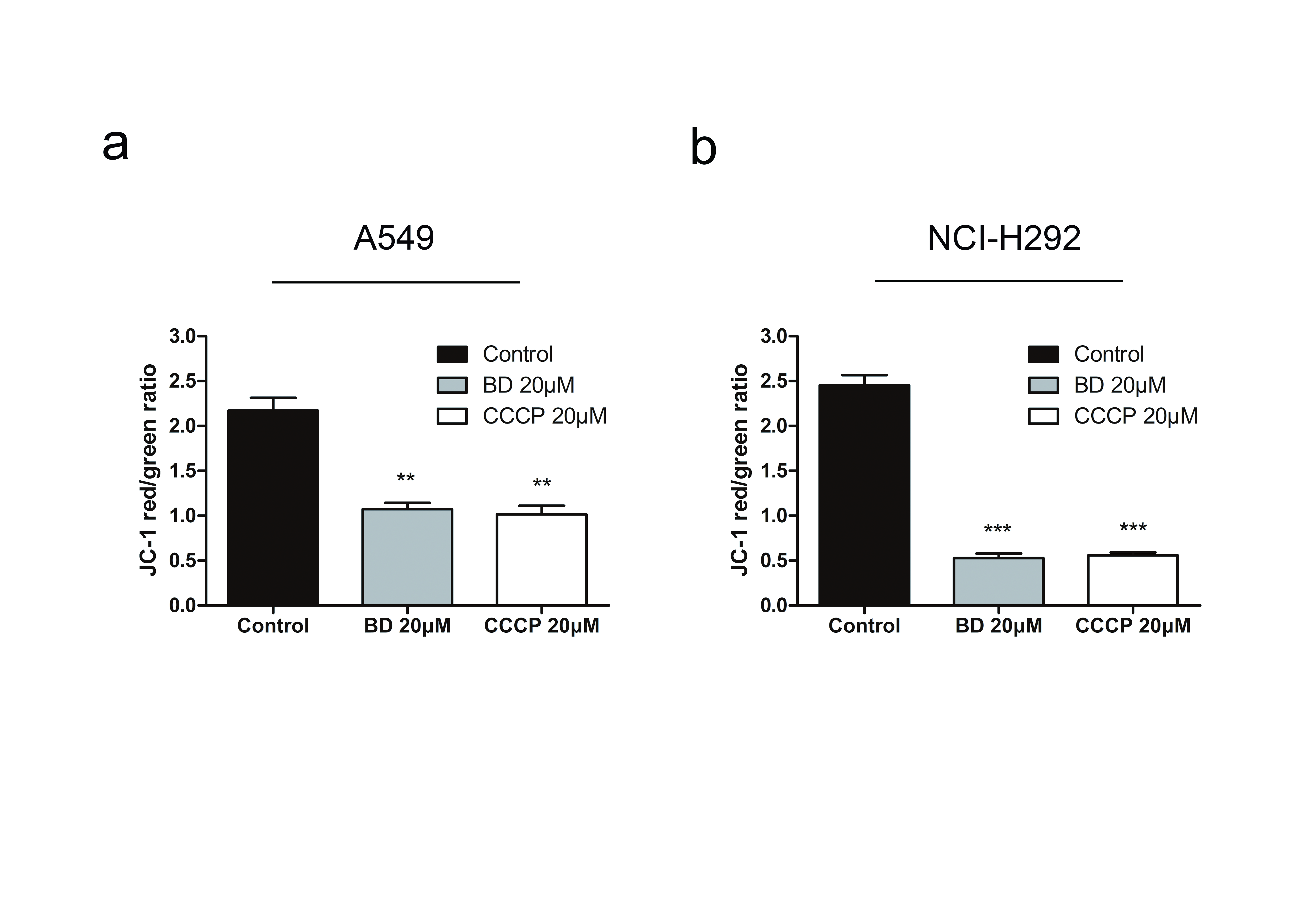

Supplement: Supplementary file 2 — Figure S2 [file 41419_2020_2317_MOESM2_ESM.tif]
