# Supplementary material for: Bruceine D induces lung cancer cell apoptosis and autophagy via the ROS/MAPK signaling pathway in vitro and in vivo
Source: Cell Death Dis. 2020 Feb 18;11(2):126. doi: 10.1038/s41419-020-2317-3 (PMC7028916; doi:10.1038/s41419-020-2317-3)
Supplement: Supplementary file 3 — Extraction and separation [file 41419_2020_2317_MOESM3_ESM.docx]

**Extraction and separation**

Air-dried seeds of B.*javanica* (10 kg) were crushed into powder. Next, 95% ethanol was employed for extraction (20 L, 4 days each, 3 times). After distillation evaporation and rotary evaporation, the crude extract (800 g) was suspended in H_2_O and successively treated with petroleum ether and CH_2_Cl_2_. The CH_2_Cl_2_ part (34.8 g) was isolated by silica column chromatography using CH_2_Cl_2_–MeOH (100:1→0:1) as a mobile phase for the gradient elution to obtain 6 fractions. Fraction 4 was further separated successively by chromatography using a silica column. The mobile phase consisted of CH_2_Cl_2_–MeOH (1:0→10:1). Finally, we obtained compound BD.
