# Supplementary material for: Bruceine D induces lung cancer cell apoptosis and autophagy via the ROS/MAPK signaling pathway in vitro and in vivo
Source: Cell Death Dis. 2020 Feb 18;11(2):126. doi: 10.1038/s41419-020-2317-3 (PMC7028916; doi:10.1038/s41419-020-2317-3)
Supplement: Supplementary file 5 — Supplementary figure Legends [file 41419_2020_2317_MOESM5_ESM.docx]

**Fig. S1: BD exhibits less toxic effect on normal human umbilical vein cell lines.**

**a b** HUVEC and EA.hy926 cells were treated with BD (2.5, 5, 10, 20, 30 and 40 µM) for 24 h and 48 h. The cell viability was evaluated by CCK-8. Values are expressed as the mean ± SD, n = 3 *p < 0.05, **p < 0.01, and ***p < 0.001 compared to control groups.

**Fig. S2: BD induces depolarization of mitochondrial membrane potential in A549 and NCI‐H292 cells.**

**a b** Quantitative analysis of the fluorescent intensity is shown in histograms**.** Values are expressed as the mean ± SD, n = 3 *p < 0.05, **p < 0.01, and ***p < 0.001 compared to control groups.
